# Use of Nonhuman Sera as a Highly Cost-Effective Internal Standard for Quantitation of Multiple Human Proteins Using Species-Specific Tryptic Peptides: Applicability in Clinical LC-MS Analyses

**DOI:** 10.1021/acs.jproteome.3c00762

**Published:** 2024-03-27

**Authors:** Geraldine Williams, Lewis Couchman, David R. Taylor, Jatinderpal K. Sandhu, Oliver C. Slingsby, Leong L. Ng, Cajetan F. Moniz, Donald J. L. Jones, Colleen B. Maxwell

**Affiliations:** † Leicester van Geest MS-OMICS Facility, Hodgkin Building, University of Leicester, Leicester LE1 9HN, United Kingdom; ‡ Leicester Cancer Research Centre, RKCSB, University of Leicester, Leicester LE2 7LX, United Kingdom; § Department of Cardiovascular Sciences and NIHR Leicester Cardiovascular Biomedical Research Unit, Glenfield Hospital, Leicester LE3 9QP, United Kingdom; ∥ Viapath Analytics, King’s College Hospital, Denmark Hill, London SE5 9RS, United Kingdom; ⊥ Department of Clinical Biochemistry, King’s College Hospital, Denmark Hill, London SE5 9RS, United Kingdom

**Keywords:** quantitation, low cost, quantitative proteomics, bottom-up proteomics, targeted proteomics, multiple reaction monitoring, selected reaction monitoring, surrogate standard, cardiovascular risk markers

## Abstract

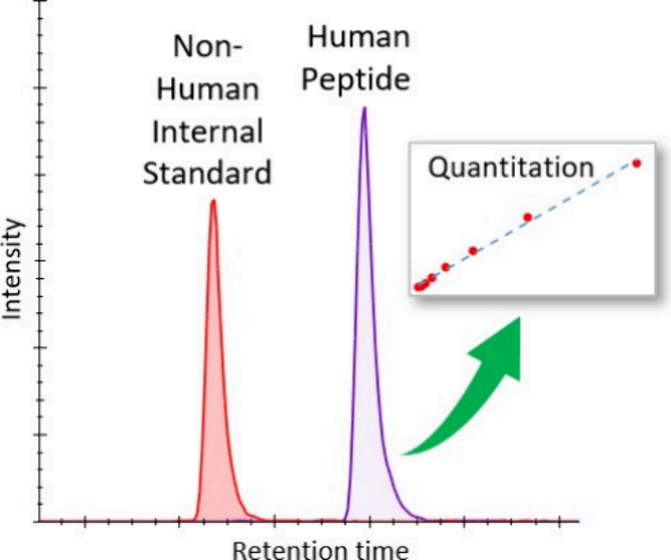

Quantitation of proteins using liquid chromatography–tandem mass spectrometry (LC-MS/MS) is complex, with a multiplicity of options ranging from label-free techniques to chemically and metabolically labeling proteins. Increasingly, for clinically relevant analyses, stable isotope-labeled (SIL) internal standards (ISs) represent the “gold standard” for quantitation due to their similar physiochemical properties to the analyte, wide availability, and ability to multiplex to several peptides. However, the purchase of SIL-ISs is a resource-intensive step in terms of cost and time, particularly for screening putative biomarker panels of hundreds of proteins. We demonstrate an alternative strategy utilizing nonhuman sera as the IS for quantitation of multiple human proteins. We demonstrate the effectiveness of this strategy using two high abundance clinically relevant analytes, vitamin D binding protein [Gc globulin] (DBP) and albumin (ALB). We extend this to three putative risk markers for cardiovascular disease: plasma protease C1 inhibitor (SERPING1), annexin A1 (ANXA1), and protein kinase, DNA-activated catalytic subunit (PRKDC). The results show highly specific, reproducible, and linear measurement of the proteins of interest with comparable precision and accuracy to the gold standard SIL-IS technique. This approach may not be applicable to every protein, but for many proteins it can offer a cost-effective solution to LC-MS/MS protein quantitation.

## Introduction

The use of liquid chromatography–tandem mass spectrometry (LC-MS/MS) has revolutionized the analysis of certain “small molecule” classes in clinical chemistry. The added specificity of MS detection over immunometric techniques or competitive binding protein assays, coupled with selective sample preparation methods and chromatographic analyses, has been exploited in many clinical laboratories. Perhaps the most notable examples are the analyses of vitamin D metabolites,^1^ steroid hormones,^2^ and therapeutic drug monitoring of immunosuppressants.^3^ LC-MS/MS is also recommended for chemical adherence testing of prescribed medication, for example in patients with apparent treatment-resistant hypertension.^4^ For clinical protein tests, on the other hand, high sensitivity immunoassays are the current gold standard.^5^ However, these have limited multiplexing ability due to cross-reactivity and can also be susceptible to lot-to-lot variation, even for commercial monoclonal antibodies.^5,6^ Peptide multiple reaction monitoring (MRM) assays typically enable more flexible assay development, better specificity, and greater multiplexing ability than immunoassays.^7^ The large scale required for biomarker validation consequently drives the growth of multiplexed targeted LC-MS/MS assays for clinical applications. High-throughput, quantitative analysis of clinically relevant proteins and peptides may therefore, in time, benefit as much as small molecule analyses from the application of LC-MS/MS.

Stable isotopes (typically ^2^H, ^13^C, ^15^N, or ^18^O) are frequently used in LC-MS/MS to provide quantitation based on a relative comparison between the light and heavy isotope forms with a number of techniques available for bottom-up proteomics analyses. One such technique is the incorporation of isotopic labels directly into the sample of interest at the protein level, followed by proteolytic digestion. Chemical labels may be introduced metabolically at the protein level: in stable isotope labeling with amino acids in cell culture (SILAC), two populations of cells are cultured: one in media containing unlabeled amino acids and one containing heavy-labeled amino acids.^8^ Labels may also be introduced to intact proteins chemically with an isotope-coded affinity tag (ICAT) that allows labeling of intact proteins on cysteine residues.^9^ Chemical labels may also be introduced to the peptides following digestion, such as the isobaric tags for relative and absolute quantification (iTRAQ) technique^11^ and tandem mass tagging (TMT),^12^ which allow the peptides to be distinguished and quantified when fragmented. With these techniques, however, there are considerable costs involved, complex sample preparation steps, difficulty ensuring close to 100% labeling efficiency, and limited multiplexing capability, which in particular limit their applicability to the clinical laboratory.

Methods of quantitation are also available that do not involve heavy labeling of the endogenous peptides or protein, but rather rely on stable isotope-labeled internal standards (SIL-ISs) to act as internal calibrants. Fully isotopically labeled analogues of the proteins of interest are preferable since this has the advantage of normalizing for potential differences resulting from proteolytic digestion efficiency. In some cases, reference materials and isotope-labeled proteins are commercially available such as for insulin, IGF-I and hepcidin,^10^ however in many cases these standards are expensive or indeed not yet commercially available, especially for large proteins with many variant forms, or novel putative biomarker proteins. Moreover, the labeled proteins should ideally incorporate tertiary and quaternary structure of the native protein, which is not always a straightforward synthetic process. Analogues for target proteins from other species,^11^ or monoclonal antibodies derived from other species,^12^ have been used with some success for protein quantitation, but rely upon production or commercial availability of the analogue protein. Thus, isotopically labeled analogues at the peptide level are often a more practical solution and are available for custom synthesis for an extensive range of targets. Known quantities of heavy-labeled peptides may be spiked into the sample and an external calibration curve constructed to determine the amount of endogenous peptide present by comparing the ratio of peak intensities of the unlabeled peptide and the SIL peptide.^13^

SIL-ISs at the peptide level are often still expensive to synthesize and procure, resulting in additional lead time in assay development and limiting the size of protein panels which can be measured. Moreover, recent global events such as Brexit,^14^ the COVID-19 pandemic, and the war in Ukraine have demonstrated that stable isotope-labeled reagents have supply chains vulnerable to disruption,^14,15^ which can lead to further delays, breaks in order fulfilment, and fluctuations in cost. These issues gain further significance in the context of the high attrition rates in translation from bench to bedside: the vast majority of proteins identified using LC-MS/MS to fill gaps in contemporary clinical pathways are still not successfully implemented as clinical tests,^16^ with much of the time expended leading to relatively poor returns. In addition, although SIL-ISs normalize for effects at the analytical level such as ionization suppression or enhancement, they do not always account for sample-to-sample or batch-to-batch variability during the digestion process. Novel approaches such as “winged peptides” or quantitative concatenated peptides (QconCAT) that are extended at the C- and/or N-terminus to incorporate cleavage sites go some way to overcoming this problem.^17^ However, digestion profiles of the shorter peptides do not typically match those of the native protein in clinical samples due to a lack of secondary and tertiary structure.^18^ The use of bovine serum albumin (BSA) as a widely available low-cost standard to normalize for sample preparation variation has previously been suggested,^19^ however, this requires further validation for use in the development of clinical protein assays.

In order to circumvent these problems, we sought to evaluate the use of fetal bovine serum (FBS), an undiluted nonhuman matrix, as a single, readily available, and extremely cost-effective IS. We demonstrate the multiplexed, simultaneous quantitation of several human peptides by utilizing the FBS-analogue peptides as nonhuman surrogate standards. We establish proof of principle with two highly abundant, clinically relevant exemplar human proteins following digestion with trypsin: vitamin D binding protein (DBP), and albumin (ALB). Serum albumin concentration has been used in the clinic for decades as a surrogate marker for total circulating protein as an indicator of nutritional status.^20^ DBP can be used to help assess levels of bioavailable vitamin D, important in a number of clinical conditions.^21^ We then extend the technique to three putative risk markers for cardiovascular disease: plasma protease C1 inhibitor (SERPING1),^22^ annexin A1 (ANXA1),^23^ and protein kinase, DNA-activated, catalytic subunit (PRKDC).^24^ We compare the common assay validation parameters of linearity, limits of detection, precision, and accuracy of the method with the gold standard SIL-IS technique and demonstrate its use in human plasma samples in a coronary artery disease (CAD) cohort.

## Methods

### Materials

Foetal bovine serum (FBS), human serum ALB, pooled human DBP, formic acid (MS grade), and acetonitrile (MS grade) were obtained from Sigma-Aldrich (Poole, U.K.). For the ALB and DBP experiments, pooled human plasma (K_2_EDTA) was purchased from Sera Laboratories (West Sussex, U.K.). SMARTDigest kits, comprising (i) digestion vials containing immobilized trypsin and (ii) SMARTDigest buffer, were from ThermoScientific (Loughborough, U.K.). Lo-Bind protein (0.5 mL) tubes were purchased from Eppendorf (Stevenage, U.K.). Glass autosampler vials (2 mL) and snap-caps were from Kinesis (St Neots, U.K.). QuanRecovery with MaxPeak 700 μL plates were obtained from Waters (Wilmslow, U.K.). Unlabeled peptides and SIL-IS labeled with ^13^C and ^15^N on the last amino acid lysine K (C6, N2) or arginine R (C6, N4) were obtained from Peptide Synthetics Peptide Protein Research Ltd. (Hampshire, U.K.) with a purity of >95% determined by HPLC. Supporting Information, Table S1 outlines the full sequences, labeling positions, and precursor *m*/*z* values for synthetic peptides. Peptide standards were stored at −20 °C as lyophilized powders until use, whereupon they were solubilized as recommended by the supplier and aliquoted into 100 μL portions for storage at −80 °C.

### Plasma Collection

Plasma was obtained from the Biomedical Research Informatics Centre for Cardiovascular Sciences (BRICCS) cohort, collected from healthy donors with informed consent under Research Ethics Committee (REC) reference: 09/H0406/114. Blood was collected by venipuncture into tubes containing ethylenediaminetetraacetic acid (K_2_EDTA) anticoagulant and stored on ice until centrifugation. The blood was centrifuged at 3200 rpm for 20 min at 4 °C using a Sorvall ST 8 Small Benchtop Centrifuge (Thermo Scientific, Loughborough, U.K.). The centrifuge was allowed to come to a halt on its own, and after stopping, the plasma was harvested from the top of the tubes, ensuring the pipet tip did not come within 3 mm of the buffy coat layer of white blood cells and platelets. The plasma was stored at −80 °C until analysis.

### Peptide Selection

Protein sequences (P02774 [VTDB_HUMAN] and Q3MHN5 [VTDB_BOVIN], P02768 [ALBU_HUMAN] and P02769 [ALBU_BOVIN], P05155 [IC1_HUMAN] and E1BMJ0 [SERPING1_BOVIN], P04083 [ANXA1_HUMAN] and P46193 [ANXA1_BOVIN], and P78527 [PRKDC_HUMAN] and E1BLB6 [PRKDC_BOVIN]) were obtained from UniProt (www.uniprot.com). For ALB and DBP, similarity analysis of the human and bovine protein sequences was performed using the Clustal Omega^25^ through the UniProt Align Tool. Sequence alignment revealed 80% sequence identity (380 identical positions, 71 similar positions) for DBP and 76.3% sequence identity (465 identical positions, 105 similar positions) for ALB. Proteins were digested (trypsin) and candidate MRM *m*/*z* pairs (precursor and product ions) generated *in silico* using Pinpoint software (version 1.3.0, Thermo Scientific, Cambridge, MA). MRM *m*/*z* pairs were chosen or excluded based on (i) species specificity, (ii) presence of isobaric parent/product ions, (iii) instrument response, and (iv) similarity of human/bovine sequences (peptide length, hydrophobicity factor/retention time). For human DBP, variant form-specific peptides^10^ were excluded. The peptides selected for ALB were LVNEVTEFAK and QTALVELVK (human) and LVNELTEFAK and QTALVELLK for the corresponding bovine ISs. The peptides selected for DBP were TSALSAK and VLEPTLK (human) and TSALSDK and ILESTLK as the corresponding bovine surrogate ISs. MRM *m*/*z* pairs, retention time, and hydrophobicity index calculated using the grand average of hydropathy (GRAVY) value for each peptide is shown in Table 1.

**Table 1:** Sequence and MRM Analysis Details for the Human Peptides and Selected Bovine Nonhuman ISs

protein	peptide^a^	species	sequence position	hydrophobicity index^b^	RT (min)	precursor ion^c^ (*m*/*z*)	CE (eV)	product ions^d^ (*m*/*z*) [ion type]
ALB^e^	LVNEVTEFAK	human	66–75	0.17	4.30	575.3	32	595.3 [y5]; 694.4 [y6]
	LVNE**L**TEFAK	bovine	66–75	0.13	4.60	582.3	32	708.4 [y6]; 837.4 [y7]
	QTALVELVK	human	550–558	0.69	4.55	500.8	28	700.5 [y6]; 771.5 [y7]
	QTALVEL**L**K	bovine	549–557	0.64	4.90	507.8	28	714.5 [y6]; 785.5 [y7]
DBP	TSALSAK	human	88–94	0.17	3.15	339.2	13	489.3 [y5]; 576.3 [y6]
	TSALS**D**K	bovine	88–94	0.56	4.00	361.2	13	533.3 [y5]; 620.3 [y6]
	VLEPTLK	human	364–370	0.56	3.95	400.2	17	587.3 [y5]; 700.4 [y6]
	**I**LES**T**LK	bovine	363–369	0.07	3.85	402.2	17	448.3 [y5]; 690.4 [y6]
SERPING1^f^	FQPTLLTLPR	human	391–400	–0.55	2.65	593.4	25	**486.3 [y4]**; 599.4 [y5]; 712.5 [y6]; 910.6 [y8]
	F**H**PT**H**LT**M**PR	bovine	365–374	–0.44	2.85	618.8	25	**504.3 [y4]**; 952.5 [y8]; 1089.6 [y9]; 617.3 [y5]
ANXA1^f^	GVDEATIIDILTK	human	59–71	0.15	3.00	694.4	16	**916.6 [y8]**; 987.6 [y9]; 815.5 [y7]; 702.4 [y6]
	GVDEATII**E**ILTK	bovine	59–71	0.15	2.70	701.4	16	**930.6 [y8]**; 716.5 [y6]; 928.5 [b9]; 1041.5 [b10]
PRKDC^f^	DQNILLGTTYR	human	3325–3335	–0.19	2.15	647.3	19	**597.3 [y5]**; 823.5 [y7]; 710.4 [y6]; 358.1 [b3]
	D**HHV**LLGTTYR	bovine	3321–3331	–0.29	1.95	656.3	19	**299.2 [y5]**; 823.5 [y7]; 922.5 [y8]; 253.1 [b2]

a Amino acid changes (in bovine sequences as relative to human sequences) are highlighted by underlining.

b Hydrophobicity index was calculated using the grand average of hydropathy (GRAVY) value.

c All reported precursor ion masses are (M + 2H)^2+^.

d All reported product ions are in the 1+ charge state.

e ALB peptides were analyzed at nonoptimum CE settings to attenuate instrument response given high concentrations of ALB relative to DBP.

f For SERPING1, ANXA1 and PRKDC, one product ion was selected as the quantifier ion, and the others served as qualifying ions. Quantifier ions are highlighted in bold.

For SERPING1, ANXA1, and PRKDC, quantotypic peptides unique to each protein within the human proteome were selected using ProteomicsDB proteotypicity rank (https://www.proteomicsdb.org/) and empirical assessment: FQPTLLTLPR for SERPING1, GVDEATIIDILTK for ANXA1, and DQNILLGTTYR for PRKDC. BLAST blastp suite was used to identify homology between the *Homo sapiens* and *Bos taurus* sequences: the bovine peptides FHPTHLTMPR for SERPING1, GVDEATIIEILTK for ANXA1 and DHHVLLGTTYR were selected. Skyline (v 22.2.0.527)^26^ with the Prosit deep learning spectral library^27^ was used to generate candidate MRM *m*/*z* pairs, shown alongside retention time and hydrophobicity index for each peptide in Table 1.

### Sample Preparation: ALB and DBP

**Figure 1:**
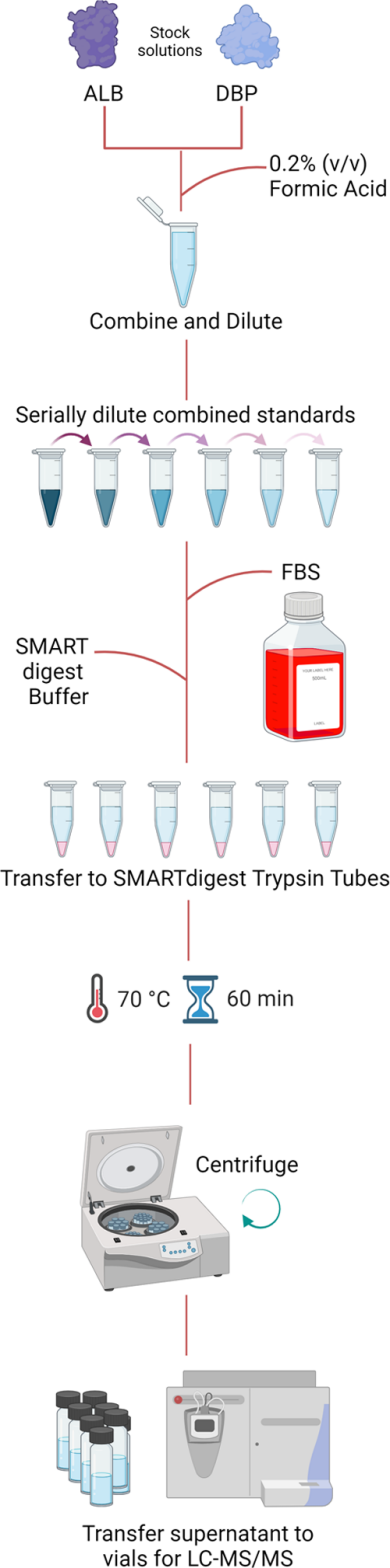
Workflow diagram for the preparation of the ALB and DBP calibration curves with a FBS nonhuman surrogate.

Sample preparation for ALB and DBP is outlined in Figure 1. Stock solutions of human ALB (100 g/L) and human DBP (1.00 g/L) were prepared in 0.2% (v/v) formic acid (FA) in deionized water (Eluent A). Combined calibrators were prepared by appropriate dilution of the human DBP and human ALB stock solutions with Eluent A, at concentrations of 10, 20, 40, 60, 80, and 100 mg/L for human DBP and of 5, 10, 20, 30, 40, and 50 g/L for human ALB. Prepared calibrators were stored at −20 °C in approximately 200 μL portions in Lo-Bind tubes prior to use. Calibrators (25 μL) were diluted with bovine serum (25 μL) and SMARTDigest buffer (450 μL) in Lo-Bind tubes and mixed by vortex (10 s). After mixing, 200 μL portions were transferred to SMARTDigest trypsin tubes. The contents of the tubes were mixed by vortex (30 s) and incubated (70 °C, 60 min). After cooling and centrifugation (13000 rpm, 5 min), the supernatant from each tube was diluted (1 + 19, *v*/*v*) with Eluent A in 2 mL autosampler vials for analysis. The six calibrators, a pooled human serum sample (without bovine serum), a bovine serum sample (without human serum or calibrator solution), and a blank sample (SMARTDigest buffer only) were each digested in duplicate, and digests were analyzed in triplicate.

### Sample Preparation: SERPING1, ANXA1, and PRKDC

**Figure 2:**
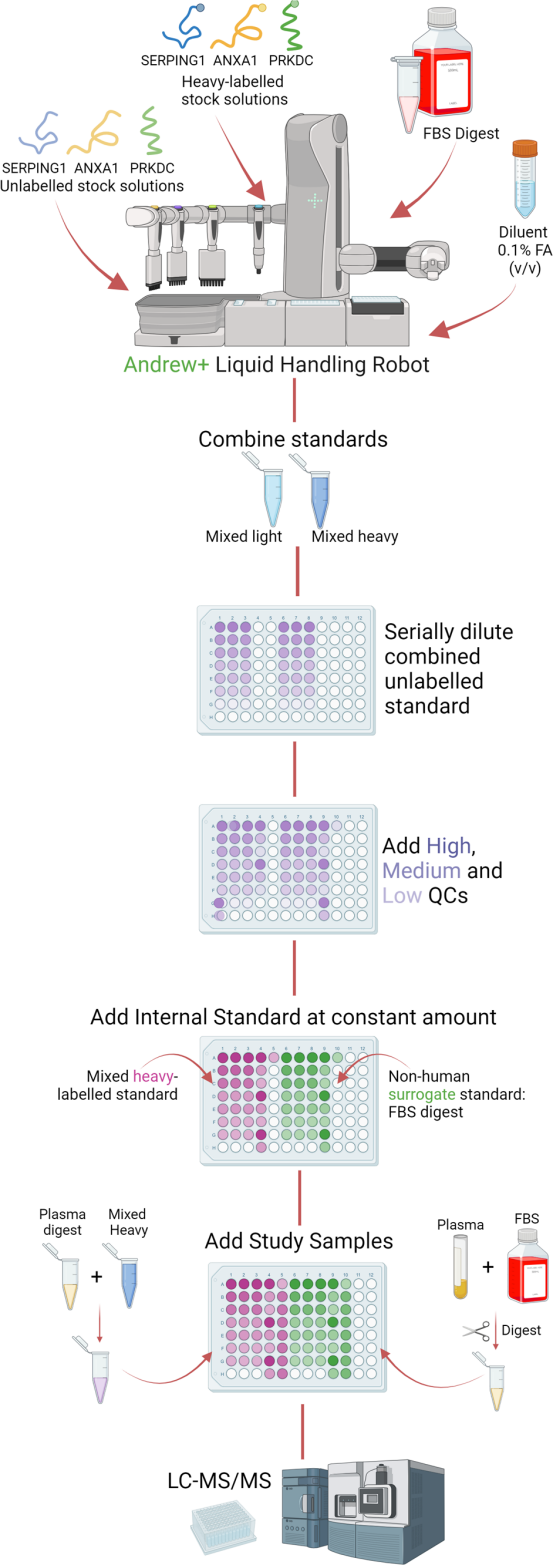
Workflow diagram for preparation of the SERPING1, ANXA1, and PRKDC calibration curves utilizing the Andrew+ liquid handling system to pipet SILS and FBS nonhuman surrogate calibrators on the same analysis plate.

Sample preparation for SERPING1, ANXA1, and PRKDC is outlined in Figure 2. Sample preparation was automated using the Andrew+ Pipetting Robot (Waters/Andrew Alliance, Milford, U.S.A.) and OneLab v1.19.1 software, following an altered protocol to enable the direct comparison with the gold standard SIL-IS technique. Each analysis involved the tandem preparation of the nonhuman surrogate IS calibration line with a SIL-IS calibration line. For each of the unlabeled peptides (FQP[...], GVD[...], and DQN[...]), fresh stock solutions of 12 pmol/μL were prepared and combined in a 1:1:1 ratio to create an unlabeled mixed stock of 4 pmol/μL. The corresponding SIL-IS peptides were prepared to 6 pmol/μL which were combined in a 1:1:1 ratio to create a SIL-IS mixed stock of 4 pmol/μL, diluted 1:20 to 100 fmol/μL with 0.1% (*v*/*v*) FA. The unlabeled mixed stock peptide mix was serially diluted 1:2 into a QuanRecovery analysis plate with 0.1% FA between 2 pmol/μL and 1 fmol/μL. IS were then added to each calibrator in a 1:1 ratio: either the SIL-IS 100 fmol/μL mixed standard to give a final concentration of 50 fmol/μL or FBS. FBS was digested following the protocol previously described by Maxwell et al.^19^ A zero calibrator containing only IS + 0.1% FA and a solvent blank containing only 0.1% FA were also prepared. Calibrators were further diluted 1:1 with 0.1% (*v*/*v*) FA.

Quality control samples were prepared on the same plate for both the SIL-IS and nonhuman surrogate at each of the following concentrations in triplicate: QC-low at 32 fmol/μL, QC-Mid at 125 fmol/μL, and QC-High at 600 fmol/μL. Each calibration line plus QCs were analyzed in triplicate across four separate analyses with an injection volume of 2 μL. A nine-point calibration curve of the following final concentration was measured: 0.49, 0.98, 1.95, 3.91, 15.63, 62.50, 250.00, 500.00, and 1000.00 fmol on column. Alongside the final calibration curve, clinical study samples were prepared using plasma from six individuals in the CAD cohort. Study samples for SIL-IS quantitation were prepared by spiking predigested plasma with mixed SIL-IS to a final concentration of 50 fmol/μL. Study samples for the nonhuman surrogate IS quantitation were prepared by combining FBS and human plasma in a 1:1 ratio prior to digestion.

### LC-MS/MS Analysis

For measurement of ALB and DBP, Eluent A was 0.2% (v/v) formic acid in deionized water and Eluent B was 0.2% (v/v) formic acid in acetonitrile. The elution gradient was from 2% B ramped to 100% B over 5 min (Aria Transcend TLX-II with Accela 600 HPLC pumps, Thermo Scientific). The flow rate was 0.50 mL/min, and the eluent was diverted to waste for (i) the first 120 s and (ii) the last 60 s of each injection. Total analysis time: 7 min. The LC column (50 × 4.6 mm i.d. (1.8 μm average particle size) Zorbax XDB-C18 (Agilent Technologies, Santa Clara, CA)) was maintained at 50 °C (Hot Pocket, Thermo Scientific). Analytes were detected using a TSQ Vantage MS/MS instrument using heated electrospray ionization (Thermo Scientific, San Jose, CA), operated in multiple reaction monitoring (MRM) mode with 0.7 FHWM resolution on both quadrupoles. Two transitions were measured per peptide, see Table 1.

For the measurement of SERPING1, ANXA1, and PRKDC LC-MS/MS analysis was performed using a Waters Acquity Premier UPLC coupled to a Xevo TQ-XS mass spectrometer. The LC was equipped with an Acquity Premier Peptide BEH C18 analytical column, 300 Å, 1.7 μm, 2.1 mm × 50 mm. Mobile phase A was H_2_O + 0.1% FA. Mobile phase B was acetonitrile (MeCN) + 0.1% FA. The seal wash was H_2_O + 10% methanol (MeOH), the weak needle wash was H_2_O + 0.1% FA, and the strong needle wash was MeCN + 0.1% FA. The flow rate was 0.6 mL/min. The autosampler temperature was 8 °C and column temperature was 40 °C. The Xevo TQ-XS was equipped with a Waters Zspray LockSpray in ESI positive mode. The cone voltage was set to 35 V and the capillary voltage was set to 0.6 kV. The 5 min LC gradient comprised a 0.5 min equilibration step at 5% B, followed by a ramp to 60% B over 3.2 min, an isocratic hold at 95% B for 0.9 min, and a re-equilibration to 5% B for 0.3 min. An optimized MRM assay was developed for FQP[...], GVD[...], and DQN[...] using MassLynx Skyline Interface (MSI) V1.2.0 for automated selection of optimum transitions, collision energy optimization, and retention time window scheduling. Data was acquired using MassLynx V4.2. The monitored transitions and collision energies and are available in Table 1.

### Method Validation

Comparison of the nonhuman surrogate IS to the gold standard SIL-IS technique was performed following Bioanalytical Method Validation guidelines as per the U.S. Food and Drug Administration (FDA)^28^ and International Council for Harmonisation of Technical Requirements for Pharmaceuticals for Human Use (ICH).^29^ The lower limit of detection (LLOD) is established per Section 7.3.2 of the ICH Topic Q2 Validation of Analytical Procedures: Text and Methodology based on LLOD = 3.3σ/*S*, where σ is the standard deviation (SD) of the y-intercepts of the regression lines and *S* is the slope.^30^ Similarly, the lower limit of quantitation (LLOQ) is estimated based on LLOQ = 10σ/*S*. Acceptance criteria for individual calibrators was that % recovery should be within ±15% of the theoretical concentrations, except at the LLOQ where they should be within ±20%. Accuracy (or bias) and precision for back-calculated concentrations should be within ±15%, except at the lower limit of quantitation (LLOQ) where the calibrators should be within ±20%. A minimum of six nonzero calibrators should meet the above criteria in each analysis. Precision is reported as %CV and accuracy (or bias) as % relative error (%RE) compared to the theoretical concentration. The regression linearity was measured using *R*^2^.

## Results

### Measurement of ALB and DBP Using Nonhuman Surrogate IS

**Figure 3:**
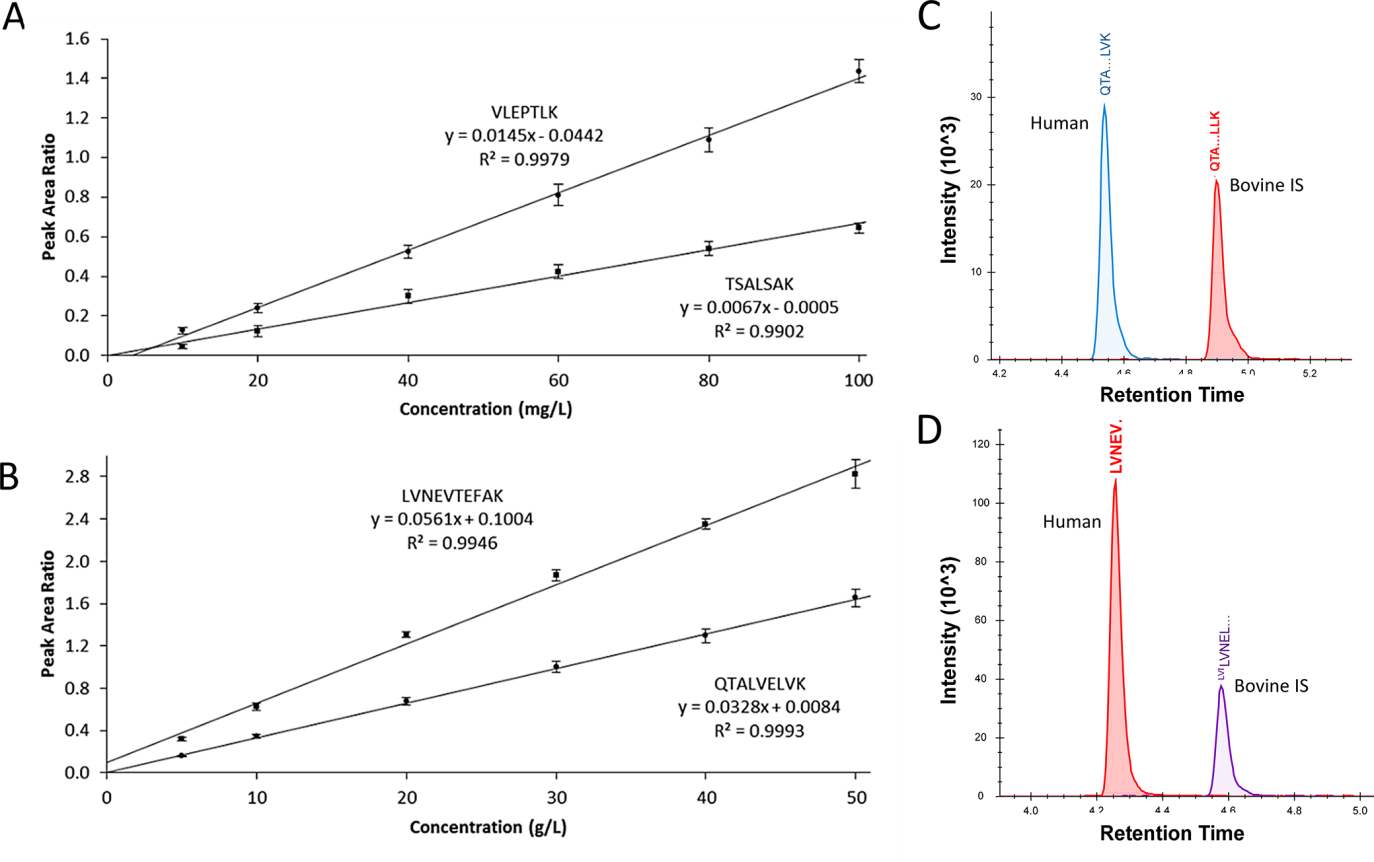
Evaluation of nonhuman surrogate IS for ALB and DBP. Calibration curves for (A) DBP peptides, and (B) ALB peptides. Plotted are the mean (±σ, *N* = 6 for each calibrator) peak area ratios (human:bovine peptide) against analyte concentration. (C) Representative chromatograms for the human ALB peptide QTA[. . .]LVK at 80 mg/L and its bovine counterpart. (D) Representative chromatograms for the human ALB peptide LVNEV[...] at 80 mg/L and its bovine counterpart.

**Figure 4:**
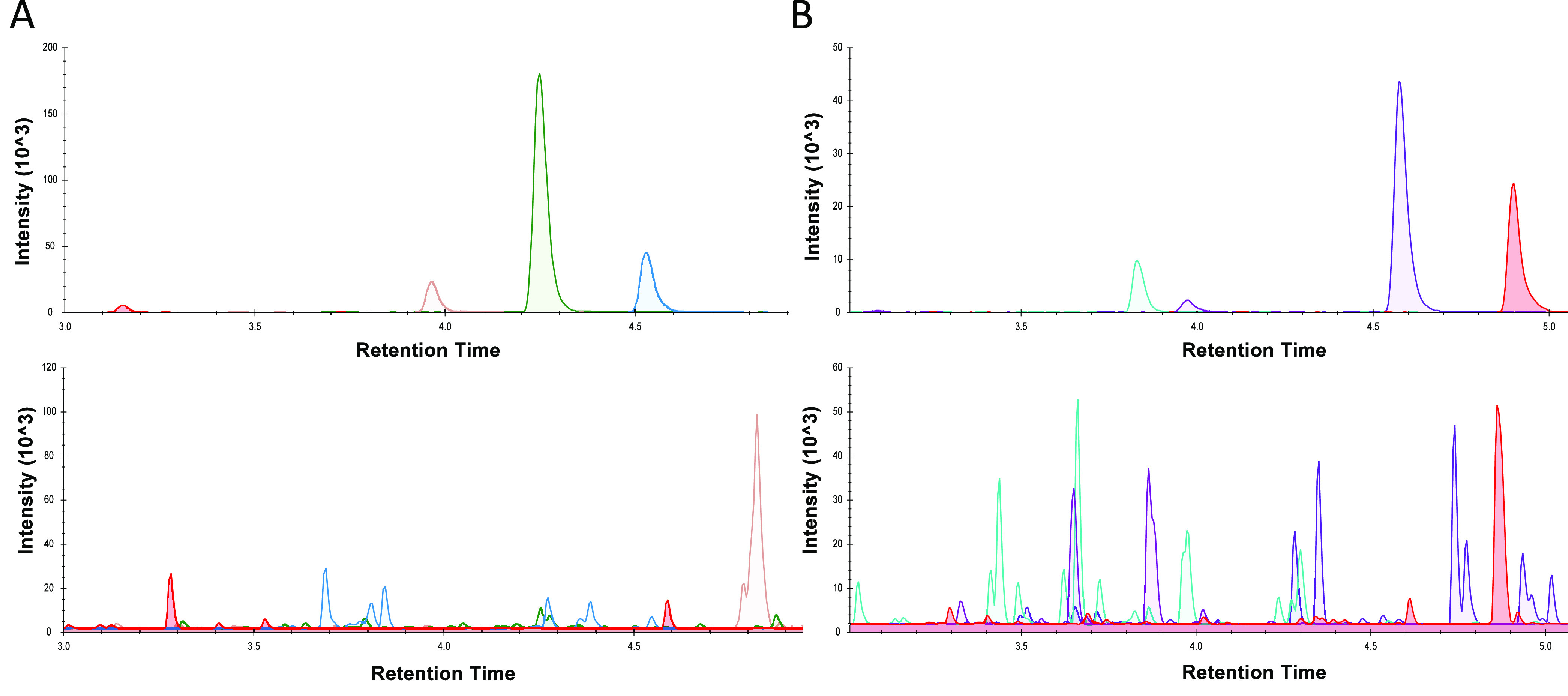
Specificity of transitions for both human analytes and bovine ISs. (A) Chromatograms showing the four human peptides for DBP (TSALSAK [1], VLEPTLK [2]) and ALB (LVNEVTEFAK [3], QTALVELVK [4]). The top panel shows the cumulative product ion peak for the two transitions chosen for each peptide when measured in human plasma. The bottom panel shows a chromatogram where the same transitions for human proteins were used to analyze bovine serum. (B) Chromatograms showing the four bovine IS peptides for DBP (TSALSDK [IS2], ILESTLK [IS1]) and ALB (LVNELTEFAK [IS3], QTALVELLK [IS4]). The upper panel shows the cumulative product ion peak for the two transitions chosen for each peptide when it was measured in bovine serum. The bottom panel shows a chromatogram where the same transitions for bovine proteins are used to analyze human plasma. Note that the scale of the upper panels in both cases is at least 1000-fold greater than that of the corresponding lower panel.

For ALB and DBP, six-point calibration curves were produced by plotting the mean peak area ratios (human/bovine IS) against analyte concentration for each of the peptides, shown in Figure 3A for DBP and Figure 3B for ALB. The calibration curves were highly linear for all four peptides (*R*^2^ > 0.99). The responses of the human ALB peptides QTA[...]LVK and LVNEV[...] with their bovine IS counterparts QTA[...]LLK and LVNEL[...] for the individual 80 mg/L calibration points are shown in Figure 3C and D, respectively. Chromatograms for LVNEV[...] and LVNEL[...] at each calibration point are shown in Supporting Information, Figure S1, illustrating that the bovine peptide peak area remains static while the human peptide changes with each calibrator. Similar hydrophobicity indices and retention times are observed for the analytes and surrogate IS, as shown in Table 1. None of the human peptides of interest were detected in the samples containing bovine serum only, and none of the bovine peptides of interest were detected in the samples containing human serum only (Figure 4), indicating high specificity. No signal was observed for the peptides of interest in the blank samples. ALB and DBP are high-abundance plasma proteins, and in addition, the absolute amounts of bovine DBP and bovine ALB in this study were not known. Thus, it was decided to extend the analysis to lower abundance proteins and perform a comparison to the gold standard SIL-IS technique.

### Extension to SERPING1, ANXA1, and PRKDC and Comparison to Gold Standard SIL-IS

**Figure 5:**
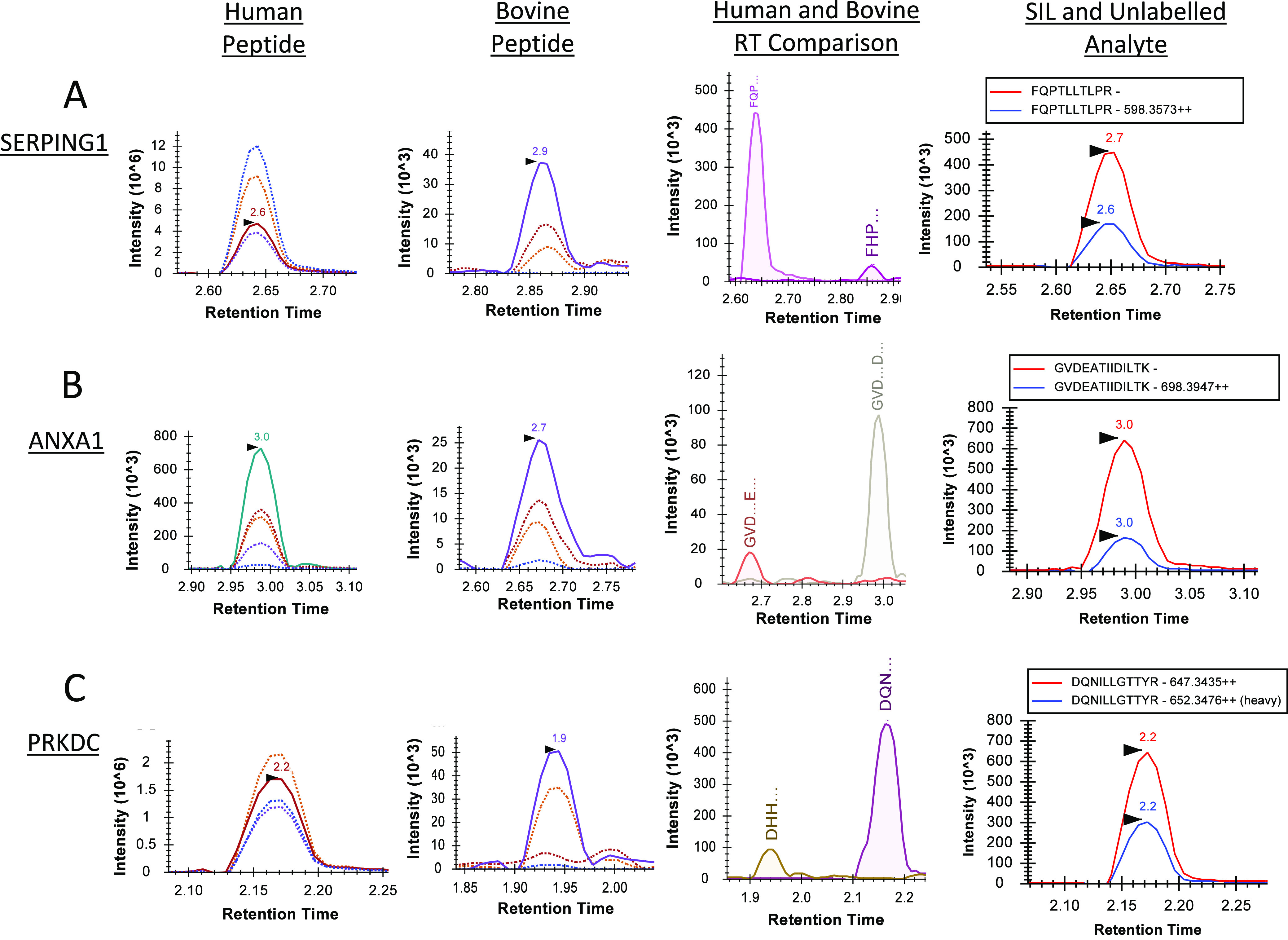
Chromatograms for each of the human peptides (first panel), FBS nonhuman surrogate IS (second panel, moving across), both analytes on the same chromatogram (third panel), and the unlabeled analyte at 125 fmol on column vs SIL-IS at 50 fmol on column for (A) SERPING1, (B) ANXA1, and (C) PRKDC. The dotted lines represent quantifier ions, while the solid lines represent the transitions used for quantitation.

For SERPING1, the bovine peptide FHP[...] was identified as a close homologue of the human peptide FQP[...] and thus was selected as a surrogate IS (Figure 5A). For ANXA1, the bovine peptide GVD[...]EILTK was utilized as a surrogate for GVD[...]DILTK (Figure 5B). For PRKDC, the bovine peptide DHH[...] was selected as a surrogate IS for human DQN[...] (Figure 5C). The human peptides and their nonhuman surrogates were measured at similar RTs, with each surrogate eluting within 0.3 min of the respective human analyte. Nine-point calibration curves were produced in four separate analyses (each *N* = 3) by plotting the peak area ratios (human/bovine IS) against analyte concentration for each set of peptides and measuring each in triplicate using LC-MS/MS. Alongside each of these analyses an equivalent calibration curve for the gold standard SIL-IS quantitation method was obtained by plotting the peak area ratio (unlabeled:SIL-IS) against analyte concentration for each of the peptides. Supporting Figure S2A shows the calibration curves obtained in Analyses 1–4 for SERPING1, **S2B** for ANXA1, and **S2C** for PRKDC. The R^2^ across all analyses, both nonhuman surrogate and SIL-IS, indicated an excellent goodness of fit of the linear regression (R^2^ > 0.99, except for GVD[ . . .] quantified by SIL-IS in which R^2^ > 0.98 in several analyses).

### Limits of Detection, Accuracy, and Precision

**Figure 6:**
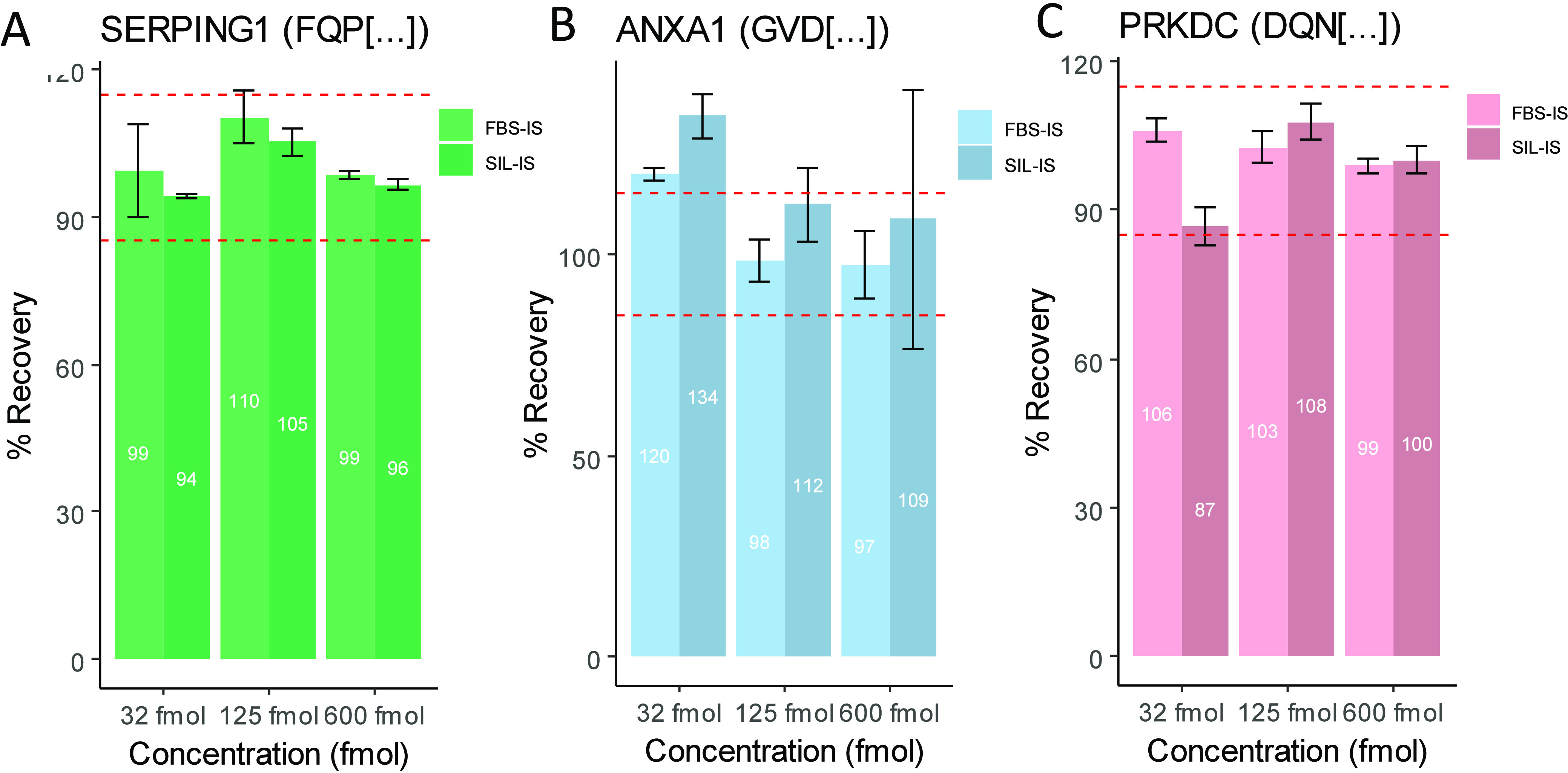
Boxplots showing mean back-calculated recovery compared to the nominal concentrations for the three QC levels across the four analyses for (A) SERPING1, (B) ANXA1, and (C) PRKDC. Error bars indicate the standard error of the mean. Red dashed lines indicate upper (115%) and lower (85%) bounds; recovery above or below these bounds may be indicative of matrix effects. Statistical significance of differences between SIL-IS and nonhuman (FBS)-IS were determined using the Wilcoxon rank signed test; significance thresholds of *p* < 0.05 and *p* < 0.01 are indicated by * and **, respectively.

Lower limits of detection and quantitation were compared for quantitation with nonhuman surrogate IS and SIL-IS. LLOD and LLOQ were calculated as described following bioanalytical method validation guidelines using σ and S values calculated across all four analyses. The results are shown in Table 2. For SERPING1 (FQP[...]) and PRKDC (DQN[...]), the LLOD and LLOQ were lower for SIL-IS quantitation, and for ANXA1 (GVD[...]) were lower for nonhuman surrogate IS quantitation. However, the differences between SIL-IS and nonhuman surrogate IS for each peptide is <1 fmol, representing comparable lower assay limits between the two techniques. Average accuracy and precision for each of the calibrators across the four analyses are summarized in Supporting Information, Table S2. Accuracy and precision were also assessed for QCs at three levels: low (32 fmol), mid (125 fmol), and high (600 fmol). Figure 6 shows the average % recovery compared to the nominal concentration for each of the QC levels for each of the analytes, with dashed lines indicating ±15% thresholds. For SERPING1 (FQP[...]), quantitation with SIL-IS slightly outperforming the nonhuman surrogate, with FBS showing poorer accuracy and precision performance at the lower assay limits. However, overall quantitation with SIL-IS and nonhuman surrogate IS were comparable, each meeting the acceptance criteria for at least six calibrators. The average % recovery was within ±15% for all QC levels, and using the Wilcoxon rank-sum test at a threshold of *p*-value < 0.05, there was no significant difference in recovery between the nonhuman surrogate and SIL-IS techniques for SERPING1.

**Table 2:** Lower Assay Limits (LLOD, Lower Limit of Detection; LLOQ, Lower Limit of Quantitation) in fmol (on Column) for Each SERPING1, ANXA1, and PRKDC for the Nonhuman Surrogate (FBS-IS) and SIL-IS Calibration Curves

	SERPING1 (FQP[...])		ANXA1 (GVD[...])		PRKDC (DQN[...])	
lower assay limits	FBS-IS	SIL-IS	FBS-IS	SIL-IS	FBS-IS	SIL-IS
LLOD (fmol)	0.66	0.91	0.48	0.35	0.24	0.35
LLOQ (fmol)	2.00	2.75	1.44	1.05	0.71	1.07

For ANXA1 (GVD[...]) using SIL-IS, on average only four calibrators met the acceptance criteria, with poor performance also seen across QC levels. Quantitation with the nonhuman surrogate IS outperformed SIL-IS, with at least six calibrators meeting the acceptance criteria as well as all QCs except for the low QC level. Both nonhuman surrogate and SIL-IS techniques had recoveries over 115% at the low QC level (low accuracy) in addition to a large measurement error (low precision) for SIL-IS at the mid and high QC levels. There was a significant difference in recovery between SIL-IS and nonhuman surrogate for ANXA1 at low and mid QC levels, with nonhuman surrogate performing better than the SIL-IS assay. For PRKDC (DQN[...]), precision and accuracy for the calibrators with SIL-IS and nonhuman surrogate IS were comparable, with the overall average accuracy and precision meeting the acceptance criteria for at least six calibrators, and a similar performance was observed across all QC levels. The average % recovery was within the threshold at all QC concentration levels; however, there was a significant difference in % recovery at the low QC level, with lower recovery in SIL-IS. Lower recovery in SIL-IS could be indicative of matrix effects such as higher levels of nonspecific binding: particularly at lower concentrations, peptides may have unpredictable adsorption to plastic and glass consumables. The addition of a complex matrix such as FBS as an adsorption competitor during the calibration curve preparation may help ameliorate these issues. Indeed, bovine serum albumin has previously been proposed as an LC-MS compatible antiadsorption diluent.^31^

### Clinical Study Samples

**Figure 7:**
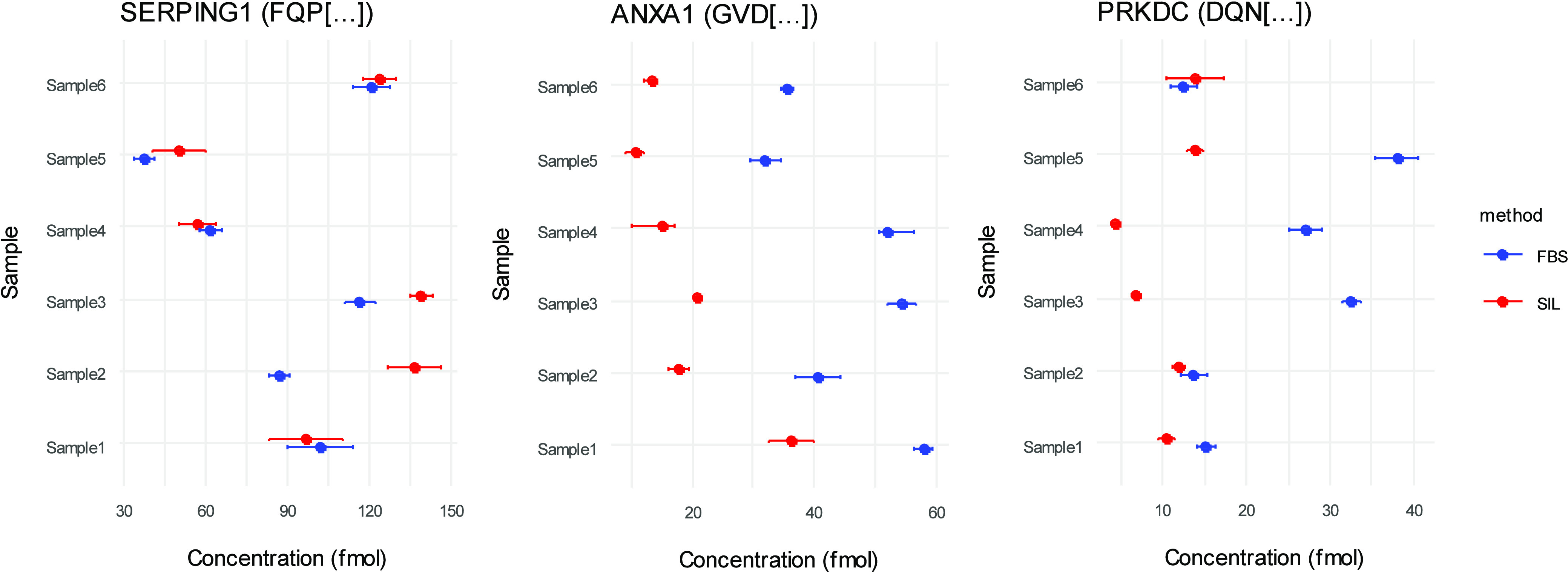
Dot plots comparing the reported concentrations (fmol on column) of 6 individual human plasma samples analyzed alongside the external calibration curves prepared in analysis 4 for (A) SERPING1, (B) ANXA1, and (C) PRKDC. The error bars represent the average ± SEM. The blue points represent FBS-IS (nonhuman surrogate), and the red points represent SIL-IS quantitation.

Alongside the final calibration curve (analysis 4), clinical study samples were prepared by using plasma from six individuals from a CAD cohort (BRICCS). A dot plot with standard error of the mean (SEM) error bars comparing back-calculated concentrations for each individual with nonhuman surrogate IS and SIL-IS is shown in Figure 7A for SERPING1, Figure 7B for ANXA1, and Figure 7C for PRKDC. A full comparison between SIL-IS and nonhuman surrogate IS alongside *p*-values for each of the comparisons and precision for the measurements is shown in Supporting Information, Table S3. For SERPING1, quantitation by SIL-IS reported endogenous concentrations across all 6 samples between ∼50 to 139 fmol on column, whereas quantitation with nonhuman surrogate concentrations ranged between ∼37 and 120 fmol. Using Wilcoxon rank-sum test for statistical significance of differences, *t* here has no significant difference between the mean (*N* = 3) calculated concentrations of the SIL-IS and nonhuman surrogate method for plasma samples 1, 4, 5, and 6, but a significantly greater (*p* < 0.01) concentration in SIL-IS-IS in samples 2 and 3. For ANXA1, quantitation by SIL-IS found endogenous concentrations across all 6 samples ranged between ∼10 to 36 fmol on column, whereas quantitation with nonhuman surrogate concentrations ranged between ∼31 to 57 fmol. There was a significant difference between the methods for all of the samples (*p* < 0.01), with quantitation by nonhuman surrogate consistently reporting higher concentrations. For PRKDC, quantitation by SIL-IS found endogenous concentrations across all 6 samples ranged between ∼3 to 13 fmol on column, whereas quantitation with nonhuman surrogate concentrations were between ∼14 to 38 fmol. There was a significant difference between quantitation methods for samples 1, 3, 4, and 5 (*p* < 0.01), with SIL-IS reporting lower calculated concentrations. Differences reported between the two techniques are to be expected; matrix effects could play a potential role, but also the inability of the SIL-IS technique to take into account sample-to-sample differences incurred at the digestion level. For the study samples, FBS and human plasma were spiked together 1:1 and digested together, thus this technique should take sample preparation differences, such as incomplete digestion, into account. The results for SERPING1 suggest that FQP[...] and FHP[...] are efficiently excised to completion by trypsin, whereas the results for ANXA1 and PRKDC are consistent with potential incomplete digestion of these proteins in some or all samples. SIL-IS may thus be under-reporting the true endogenous concentrations as a result, with quantitation with FBS normalizing for these digestion inconsistencies. Despite the differences reported between techniques for protein quantitation in plasma, we demonstrate high levels of accuracy and precision for both methods of quantitation, with the exception of GVD[...] SIL-IS, as shown in Supporting Information, Table S2.

## Discussion

Despite perhaps seeming counterintuitive to add a second highly complex matrix to human samples for quantitation, this study shows the potential of using nonhuman peptides from an undiluted matrix as an extremely cost-efficient IS for simultaneous quantitation of multiple human proteins. In the work described, we have used selected tryptic peptides derived from bovine serum for quantitation of clinically relevant proteins by LC-MS/MS. The chosen peptides for both species were of similar length, had similar predicted chromatographic properties, were from corresponding positions within the entire protein sequences, and overall possessed high levels of sequence homology between species.

We demonstrate that external calibration lines produced using nonhuman surrogate ISs can perform comparably in terms of accuracy, precision, and limits of detection compared to the gold standard SIL-ISs technique and can be used to measure protein levels in human plasma using a CAD cohort as a case study. Indeed, in the case of ANXA1, the use of FBS as a nonhuman IS provided an alternative technique for quantitation when SIL-ISs failed to meet assay validation acceptance criteria. We have established excellent linearity of the technique for constructing external calibration curves using two different sample preparation techniques (one for ALB and DBP, and another for SERPING1, ANXA1, and PRKDC), demonstrating the robustness of the technique to different bottom-up proteomics protocols, adaptable to the protein of interest, and the nature of the experiment.

There are challenges associated with this strategy, however; the surrogate peptide sequences selected must be distinct from the human peptide such that they can be differentiated by *m*/*z*, but even minor changes in the sequence of a peptide can alter the efficiency of trypsinolysis and the most abundant charge state. For example, the bovine PRKDC peptide DHHVLLGTTYR differs from the human equivalent DQNILLGTTYR at the N-terminus, with the double histidine resulting in a different local charge context and potentially leading to a different likelihood of trypsinolysis. Thus, similarly to the QConCAT strategy, the internal standard may have subtle differences in proteotypicity and gas phase ion chemistry compared to the endogenous peptide. Indeed, in any strategy involving peptide-level protein quantitation with internal standards, careful selection of the peptide used is recommended to ensure accurate quantitation of the target protein. In this case, steps should be taken to ensure that both the target peptide in humans and the nonhuman surrogate represent proteotypic and quantotypic peptides to the fullest possible extent.

As a first pass, hard filter criteria such as length, lack of variable post-translational modifications (PTMs), and lack of dibasic content may be considered following the same guidelines as those recommended by Hubbard et al. for the selection in QconCAT.^17^ In addition to this, databases are available which enable researchers to select highly detectable peptides based on experimental data from endogenous proteins, such as ProteomicsDB proteotypicity rank^32^ and the PeptideAtlas “Predicted Highly Observable Peptides” feature.^33^ However, it is worth noting that other species including *Bos taurus* are typically less annotated in these databases than *Homo sapiens* and more common model organisms such as *Mus musculus*. In these cases, methods based on statistical inference and machine-learning algorithms which have been developed to predict MS detectability of a peptide may be employed, including Absolute Protein Expression (APEX) profiling,^34^ PeptideSieve,^35^ CONSeQuence,^36^ and Advanced Proteotypic Peptide Predictor (AP3).^37^ More recently, AlacatDesigner developed for the QconCAT strategy has become available,^38^ as well as Typic, which combines data-dependent acquisition (DDA) input and downloads from public repositories to rank proteotypic peptides.^39^

With these considerations in mind, the use of a nonhuman surrogate has several key advantages compared to other methods of quantitation in addition to cost-efficiency. Rather than using multiple nonhuman or isotopically labeled analogue ISs for each target analyte within a single assay, use of the entire nonhuman matrix means that this approach can provide a single, highly multiplexable, almost “universal” IS for a wide array of proteins, provided of course that unique, species-specific peptides are produced during the digestion step. The use of FBS for quantitation also represents a sustainable solution; by taking advantage of a readily available animal waste product already used in many laboratories, there is a reduced need for the synthesis and shipment of custom SIL peptides for screening candidates in clinical viability or biomarker studies. In addition, for SERPING1, ANXA1, and PRKDC, we have demonstrated the potential for high throughput automation of the technique with the Andrew+ liquid handling system.

This method of quantitation could also feasibly be adopted for quantitation of proteins in studies where another species is the target, for example, in studies involving proteomic analysis of mouse models, provided there is suitable homology between *Mus musculus* and *Bos taurus* for the protein of interest. Additionally, for the analysis of some proteins where suitable surrogates are not present in the bovine proteome owing either to total homology or low homology to *Homo sapiens*, nonhuman matrices other than bovine serum (e.g., porcine, murine) may be useful or necessary. Although these matrices may be harder to obtain, we actively recommend sharing waste blood and tissue from other species, where ethically appropriate, as a sustainable use of laboratory animal waste. For other species and other target proteins where the abundance of the protein/peptide target varies, the approach may be adapted, for example, by dilution of one of the matrices to obtain similar instrument responses for accurate quantitation or by incorporation of immodepletion of one or both matrices.

Further assay validation should be performed prior to implementation of the technique for larger human cohort studies, including assessment of storage stability, assessment using additional matrices where applicable, such as cell lysate and tissue specimens, and further benchmarking against gold standard techniques of protein quantitation. Researchers should also be aware that use of FBS from different lots may result in batch-to-batch variation and should perform a batchwise test of reproducibility if using different lots within studies.

## Conclusion

The approach described herein for human protein quantitation is highly cost-efficient, multiplexable, and sustainable. For assays in which many proteins are to be analyzed simultaneously and where isotope-labeled proteins or peptides are either unavailable or are prohibitively expensive, this method offers a simple solution that makes use of a byproduct of the meat industry, which is already available in many laboratories. Particularly in the early stages of method development and validation, this technique will enable researchers to establish and refine their analytical techniques and perform screening of large numbers of clinically relevant protein biomarker candidates without incurring significant costs. This could ultimately help to increase the capacity for biomarker validation at the bottleneck in the pipeline, tackling the challenging task of balancing cost with the high likelihood of biomarkers failing clinical validation.

## Data Availability

Raw MS data files and transition lists are deposited at The PeptideAtlas SRM Experiment Library (PASSEL), unique identifier PASS05846 and password FK4453rp.

## Supporting Information Available

Table S1: Synthetic peptides used for SERPING1, ANXA1, and PRKDC; Table S2: Assay validation for SERPING, ANXA1, and PRKDC; Table S3: Average concentrations of each of the individual plasma samples using nonhuman surrogate IS and SIL-IS for SERPING1, ANXA1, and PRKDC; Figure S1: Representative chromatograms for one of the human ALB peptides and its bovine counterpart; Figure S2: Nonhuman surrogate IS vs SIL-IS calibration curves over the same concentration range for SERPING1 ANXA1, and PRKDC peptides (PDF)

## Abbreviations

LC-MS/MS: liquid chromatography-tandem mass spectrometry
MRM: multiple reaction monitoring
IS: internal standard
SIL-IS: stable isotopically labeled internal standard
FBS: fetal bovine serum
DBP: vitamin D binding protein
ALB: albumin
SERPING1: plasma protease C1 inhibitor
ANXA1: annexin A1
PRKDC: protein kinase
DNA-activated: catalytic subunit
