# Supplementary material for: Use of Nonhuman Sera as a Highly Cost-Effective Internal Standard for Quantitation of Multiple Human Proteins Using Species-Specific Tryptic Peptides: Applicability in Clinical LC-MS Analyses
Source: J Proteome Res. 2024 Mar 27;23(8):3052–63. doi: 10.1021/acs.jproteome.3c00762 (PMC11301776; doi:10.1021/acs.jproteome.3c00762)
Supplement: Supplementary file 1 — pr3c00762_si_001.pdf [file pr3c00762_si_001.pdf]

Supporting Information

Use of Non-human Sera as a Highly Cost-Effective Internal Standard for Quantitation of Multiple Human Proteins using Species-Specific Tryptic Peptides: Applicability in Clinical LC-MS Analyses

*‡Geraldine Williams[1,3], ‡Lewis Couchman[4], David R. Taylor[5], Jatinderpal K. Sandhu[1,3], Oliver C. Slingsby[1,3], Leong L. Ng[1,3], Cajetan F. Moniz[5], Donald J.L. Jones[1,2,3], Colleen B. Maxwell[1,3]**

1. Leicester van Geest multi-OMICS Facility, Hodgkin Building, University of Leicester, Leicester, LE1 9HN, UK
2. Leicester Cancer Research Centre, RKCSB, University of Leicester, Leicester, United Kingdom, LE2 7LX, UK
3. Department of Cardiovascular Sciences and NIHR Leicester Cardiovascular Biomedical Research Unit, Glenfield Hospital, Leicester, LE3 9QP, UK
4. Analytical Services International Ltd., St. George's – University of London, Cranmer Terrace, London, SW17 0RE, UK
5. Department of Clinical Biochemistry, King's College Hospital, Denmark Hill, London, SE5 9RS, UK

‡ Equal contribution, *Corresponding author, Email: cbm11@leicester.ac.uk.

Contents

- **Table S1**. Synthetic peptides used for SERPING1, ANXA1 and PRKDC. [Page 2]
- **Figure S1**. Representative chromatograms for one of the ALB peptides (human and bovine). [Page 3]
- **Figure S2**. Non-human surrogate IS *vs.* SIL-IS calibration curves over the same concentration range for SERPING1 ANXA1, and PRKDC peptides [Pages 4-6]
- **Table S2.** Assay validation for SERPING, ANXA1 and PRKDC. [Page 7]
- **Table S3.** Average concentrations of each of the individual plasma samples using non-human surrogate IS and SIL-IS for SERPING1, ANXA1, and PRKDC. [Page 8]

Protein	Peptide	Label	Sequence and Label Position[(A)]	Precursor Ion (*m/z*)[(B)]
**SERPING1**	FQPTLLTLPR	Unlabelled	FQPTLLTLPR	593.4
		Heavy-labelled on Arginine	FQPTLLTLP**R**	598.4
**ANXA1**	GVDEATIIDILTK	Unlabelled	GVDEATIIDILTK	694.4
		Heavy-labelled on Lysine	GVDEATIIDILT**K**	698.4
**PRKDC**	DQNILLGTTYR	Unlabelled	DQNILLGTTYR	647.3
		Heavy-labelled on Arginine	DQNILLGTTY**K**	652.3

Table S1. Synthetic peptides used for SERPING1, ANXA1 and PRKDC, including heavy label used, the position of the label in each sequence, and the precursor ion *m/z* for each peptide. (**A**) The position of the heavy label, where appropriate, is indicated in the colour and underlined. (**B**) *m/z* for the $(M+H)^{2+}$ precursors.

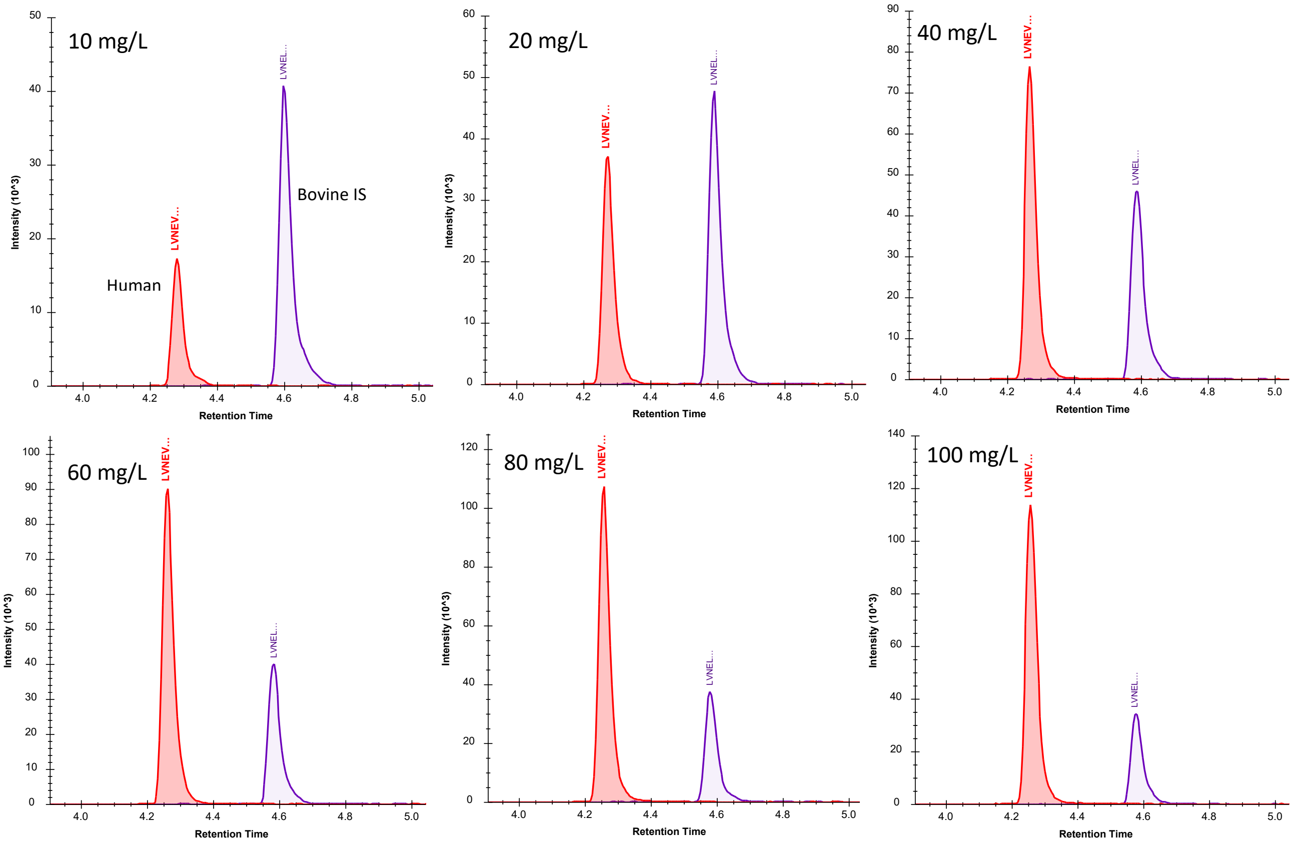

Figure S1. Representative chromatograms for one of the human ALB peptides and its bovine counterpart. The six chromatograms show the two overlaid cumulative product ion peaks for the LVNEVTEFAK ALB peptide and its bovine counterpart (LVNELTEFAK). The six panels constitute the calibration curve ranging from 10 mg/L to 100 mg/L. Inset to the first panel is the full 7 min chromatogram to show the high level of specificity obtained for this assay. Note that the bovine signal remains static whilst the human ALB peptide increases according to the concentration.

A SERPING1 FQPTLLTLPR

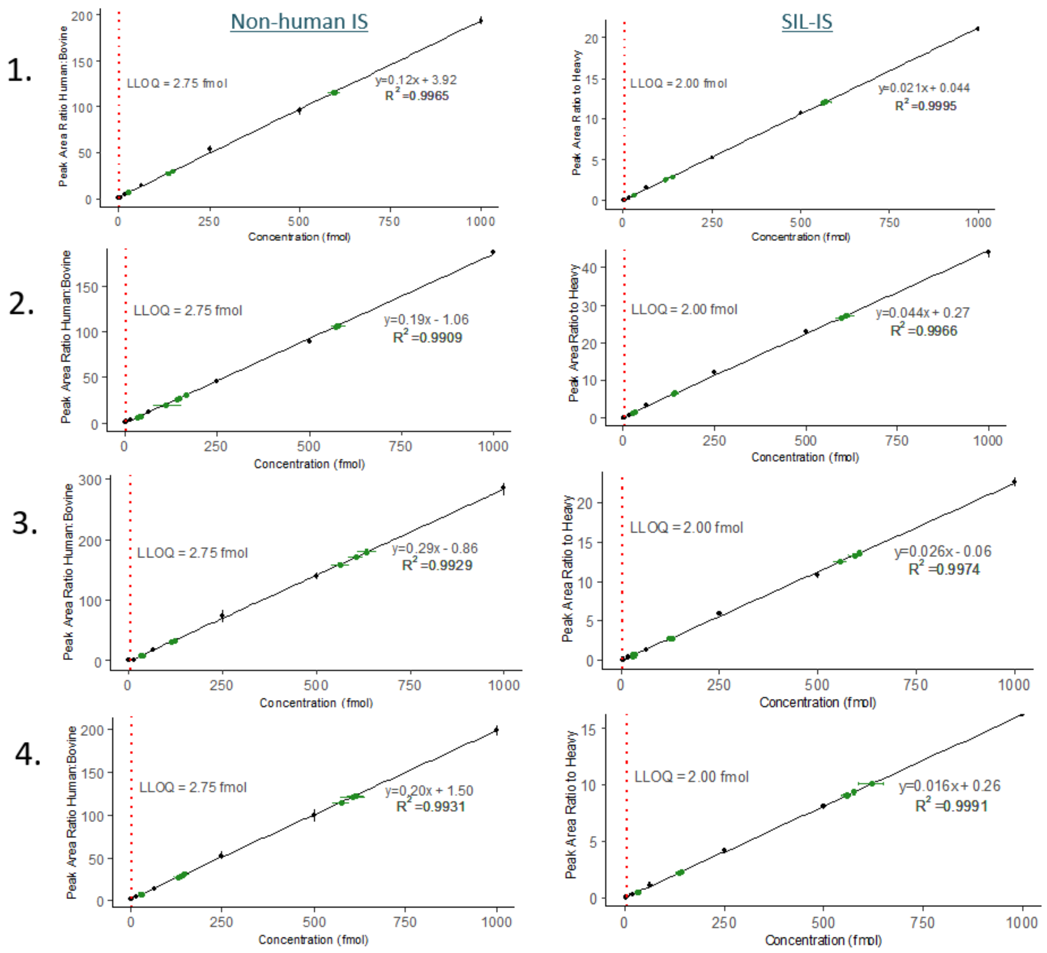

B ANXA1 GVDEATIIDILTK

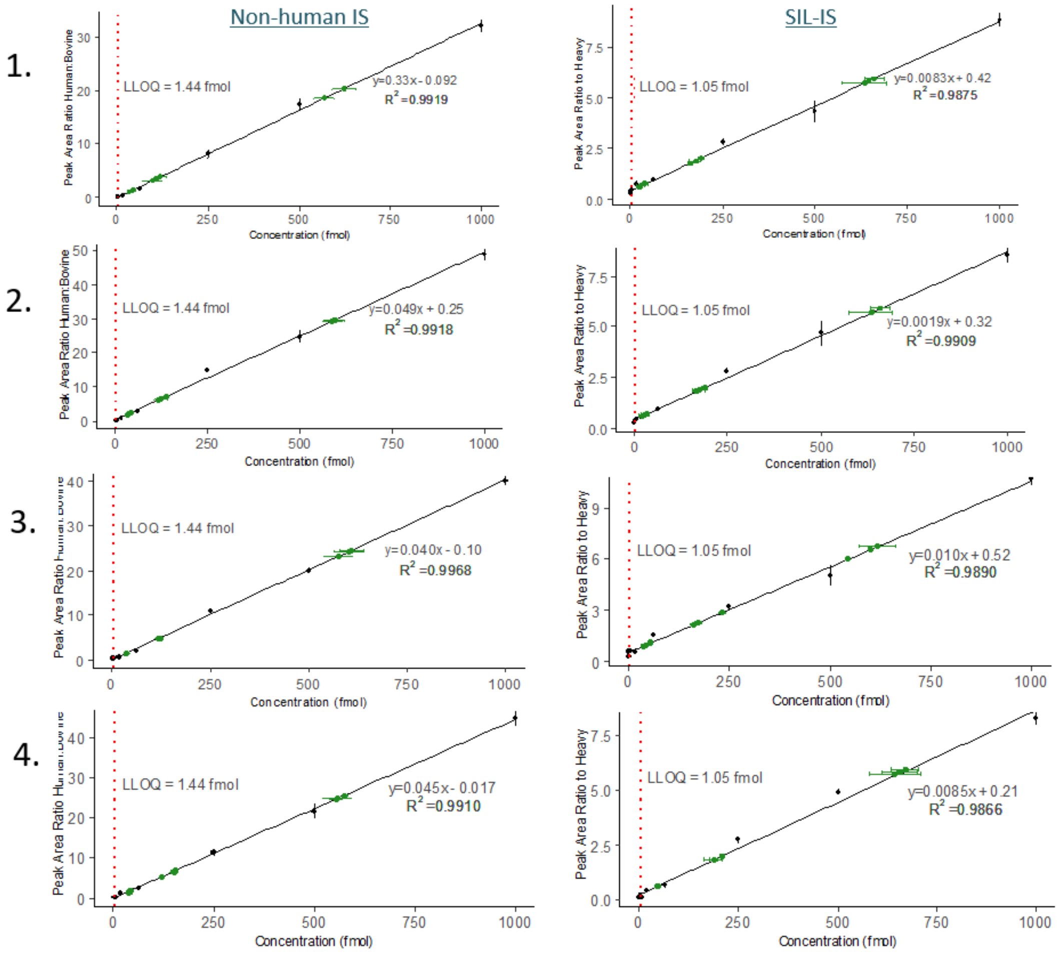

C   PRKDC DQNILLGTTYR

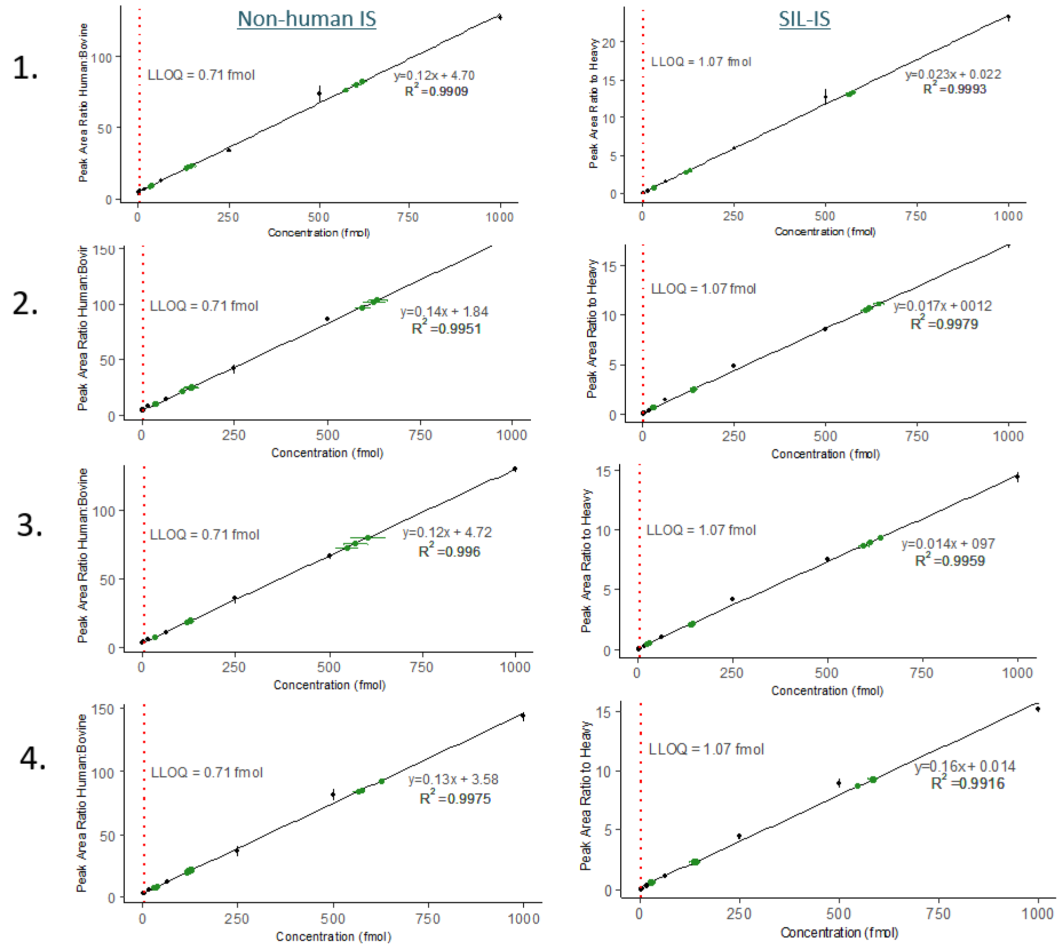

Figure S2: Non-human surrogate IS (left panel) *vs.* SIL-IS (right panel) calibration curves over the same concentration range (0.5 – 1000 fmol on column) for (**A**) SERPING1, (**B**) ANXA1, and (**C**) PRKDC peptides for each separate analyses 1 - 4. Plotted are the mean (± standard error of the mean, N = 3 for each calibrator) peak area ratios against analyte concentration. Calibrators are plotted in black, with high (600 fmol), mid (125 fmol) and low (32 fmol) plotted in green. LLOQ is indicated by the dashed red line. Calibration lines are annotated with their linear regression equations and goodness of fit (R^2).

SERPING1 (FQP[...])	Non-human Surrogate								SIL-IS							
Calibrator (fmol)	1	2	3	4	Average (A)	Standard Deviation (σ)	Precision (% CV)	Accuracy (% RE)	1	2	3	4	Average (A)	Standard Deviation (σ)	Precision (% CV)	Accuracy (% RE)
0.49	0.33*	0.26*	0.70*	0.59	0.47	0.21	44.61*	-3.66	0.57	0.48	0.59	0.41	0.51	0.08	16.30*	4.43
0.98	1.05	0.82*	1.38*	1.36*	1.16*	0.27	23.11*	18.30*	0.71*	1.07	1.30*	1.04	1.03	0.24	23.46*	5.63
1.95	2.06	2.01	3.96*	2.35*	2.60*	0.92	35.53*	32.89*	1.74	2.82*	1.76	1.60	1.98	0.57	28.69*	1.36
3.91	3.91	4.00	4.79	4.08	4.20	0.40	9.51	7.62	4.02	3.66	4.85*	4.05	4.15	0.50	12.16	6.32
15.63	15.26	17.30	14.17	15.07	15.45	1.32	8.56	-1.12	13.93	14.01	18.74*	16.27	15.74	2.28	14.47	0.71
62.50	70.16	67.64	65.83	60.07	65.93	4.29	6.51	5.48	70.17	69.82	59.90	67.85	66.93	4.80	7.17	7.09
250.00	272.27	247.24	265.49	259.73	261.18	10.61	4.06	4.47	248.06	267.31	263.17	256.54	258.77	8.40	3.25	3.51
500.00	489.33	485.00	492.28	495.80	490.60	4.58	0.93	-1.88	509.62	515.63	478.96	497.53	500.44	16.17	3.23	0.09
1000.00	999.36	1007.79	999.98	999.88	1001.75	4.03	0.40	0.18	995.26	987.52	1007.36	999.30	997.36	8.27	0.83	-0.26

ANXA1 (GVD[...])	Non-human Surrogate								SIL-IS							
Calibrator (fmol)	1	2	3	4	Average (A)	Standard Deviation (σ)	Precision (% CV)	Accuracy (% RE)	1	2	3	4	Average (A)	Standard Deviation (σ)	Precision (% CV)	Accuracy (% RE)
0.49	0.51	0.40	0.43	0.46	0.45	0.05	11.29	-8.12	0.77*	0.86*	0.30*	0.69*	0.65*	0.25	37.82*	33.56*
0.98	1.33*	1.19	1.50*	0.92	1.24*	0.25	19.97*	26.62*	2.19*	1.55*	1.06	1.40*	1.55*	0.47	30.59*	58.63*
1.95	2.68*	2.44*	2.29	2.26	2.41*	0.19	7.96	23.63*	2.94*	2.63*	2.63*	2.46*	2.66*	0.20	7.65	36.42*
3.91	3.99	3.85	5.00*	4.37	4.30	0.51	11.93	10.29	8.20*	5.90*	5.42*	4.62	6.03*	1.54	25.44*	54.74*
15.63	13.34	16.11	17.70	18.87*	16.51	2.40	14.51	5.64	20.33*	34.17*	13.89	21.05*	22.36*	8.51	38.05*	43.09*
62.50	52.16	54.73	51.17*	54.44	53.13	1.74	3.27	-15.01	64.69	61.80	85.21*	49.01	65.18	14.99	23.00*	4.28
250.00	249.53	298.17*	271.84	255.80	268.21	21.70	8.07	7.53	245.51	284.96	262.71	294.43*	271.90	22.05	8.11	8.76
500.00	533.25	498.63	496.39	488.58	504.21	19.83	3.93	0.84.	511.62	467.12	390.77*	552.48	480.50	69.24	14.41	-3.90
1000.00	984.40	989.30	997.25	1004.65	993.90	8.91	0.90	-0.61	995.16	1006.21	1011.46	1028.36	1010.30	13.82	1.37	1.03

PRKDC (DQN[...])	Non-human Surrogate								SIL-IS							
Calibrator (fmol)	1	2	3	4	Average (A)	Standard Deviation (σ)	Precision (% CV)	Accuracy (% RE)	1	2	3	4	Average (A)	Standard Deviation (σ)	Precision (% CV)	Accuracy (% RE)
0.49	0.19*	0.49	0.70*	0.74*	0.53	0.25	47.47*	8.35	0.47	0.43	0.49	0.25*	0.41	0.11	26.53*	-16.42*
0.98	0.19*	1.59*	1.45*	1.29	1.13	0.64	56.62*	15.65*	1.13*	0.88	0.86	0.86	0.94	0.13	14.12	-4.13
1.95	2.99*	2.86*	3.27*	2.50	2.90*	0.32	11.04	48.70*	1.95	2.03	1.73	1.14*	1.71	0.40	23.36*	-12.33
3.91	4.44	4.39	4.27	3.45	4.14	0.46	11.17	6.06	3.01*	3.87	3.76	5.30*	3.99	0.96	24.04*	2.19
15.63	14.85	19.56	15.92	14.26	16.15	2.38	14.72	3.34	13.91	15.00	12.10*	11.58*	13.15*	1.59	12.07	-15.86
62.50	61.18	62.60	59.41	64.45	61.91	2.14	3.46	-0.95	65.64	74.48	64.26	59.63	66.00	6.21	9.41	5.60
250.00	232.60	238.08	254.28	234.27	239.81	9.92	4.14	-4.08	255.34	277.82	280.55	273.03	271.69	11.33	4.17	8.67
500.00	548.66	525.38	501.66	548.25	530.99	22.38	4.21	6.20	546.29	496.00	512.92	563.49	529.68	30.74	5.80	5.94
1000.00	980.14	990.19	998.32	979.71	987.09	8.92	0.90	-1.29	997.85	994.46	985.92	962.96	985.30	15.72	1.59	-1.47

Table S2: Assay validation for SERPING, ANXA1 and PRKDC, calculated for each calibration point across four separate triplicate analyses for the non-human surrogate IS (left) and SIL-IS (right). Calibrators which do not meet the acceptance criteria for Precision and Accuracy are highlighted with *. (**A**) The average is the average back-calculated concentration of each calibrator across the analyses.

SERPING1 (FQP[...])	Non-human Surrogate			SIL-IS			FBS *vs.* SIL-IS
Sample	Average [(A)]	Standard Deviation (σ)	Precision (% CV)	Average [(A)]	Standard Deviation (σ)	Precision (% CV)	p-value [(B)]
1	101.76	12.10	11.90	96.89	13.34	13.77	0.66
2	87.06	3.67	4.22	136.59	9.94	7.27	0.0071**
3	116.33	5.70	4.90	139.04	4.01	2.89	0.0067**
4	61.68	4.00	6.48	56.78	6.67	11.74	0.35
5	37.80	3.84	10.15	50.18	9.51	18.94	0.14
6	120.78	6.56	5.43	123.59	5.83	4.72	0.61

ANXA1 (GVD[...])	Non-human Surrogate			SIL-IS			FBS *vs.* SIL-IS
Sample	Average [(A)]	Standard Deviation (σ)	Precision (% CV)	Average [(A)]	Standard Deviation (σ)	Precision (% CV)	p-value [(B)]
1	57.96	1.66	2.87	36.35	3.74	10.30	0.0039**
2	40.74	3.68	9.03	17.74	1.73	9.73	0.0028**
3	54.57	2.35	4.30	20.90	0.11	0.54	0.0016**
4	52.02	3.02	5.81	15.05	3.59	23.89	0.00016***
5	31.97	2.62	8.20	10.50	1.49	14.15	0.00088***
6	35.55	1.09	3.06	13.37	1.04	7.74	0.000014***

PRKDC (DQN[...])	Non-human Surrogate			SIL-IS			FBS *vs.* SIL-IS
Sample	Average [(A)]	Standard Deviation (σ)	Precision (% CV)	Average [(A)]	Standard Deviation (σ)	Precision (% CV)	p-value [(B)]
1	15.16	1.16	7.68	10.44	0.93	8.91	0.00064***
2	13.64	1.55	11.37	11.89	0.77	6.43	0.18
3	32.53	1.11	3.41	6.61	0.35	5.26	0.00021***
4	27.08	1.84	6.81	4.16	0.33	8.01	0.0017**
5	38.01	2.66	7.01	13.90	0.99	7.10	0.0016**
6	12.47	1.68	13.50	13.78	3.41	24.74	0.59

Table S3: Average concentrations of each of the individual plasma samples quantitated in Analysis 4 using non-human surrogate IS (left) and SIL-IS (right) for SERPING1, ANXA1, and PRKDC. The standard deviation and precision are also shown. (**A**) The average is the mean (N=3) back-calculated concentration of each calibrator across the analyses. (**B**) The final column shows the p-values from a comparison of the means between the two techniques for each sample, calculated using Wilcoxon rank-sum test. Significance thresholds are: of $p<0.05$ *, $p<0.01$ **, $p<0.001$ ***.
